# Daily Pain and Activity Interference Trajectories Following Primary Motor Cortex rTMS for Endometriosis-Associated Chronic Pelvic Pain: A Prospective Real-World Cohort Study

**DOI:** 10.3390/life16101686

**Published:** 2026-10-09

**Authors:** Marius Nicolae Popescu, Cristina Popescu, Claudiu Căpeț, Simona Elena Săvulescu, George-Alexandru Filipescu

**Affiliations:** 1 Department of Physical and Rehabilitation Medicine, Elias Emergency University Hospital, Carol Davila University of Medicine and Pharmacy, 020021 Bucharest, Romania; marius.popescu@umfcd.ro (M.N.P.); simona.savulescu@umfcd.ro (S.E.S.); 2 Clinic of Physical and Rehabilitation Medicine, Elias Emergency University Hospital, 011461 Bucharest, Romania; 3 Department of Oncologic Dermatology, Elias Emergency University Hospital, Carol Davila University of Medicine and Pharmacy, 020021 Bucharest, Romania; 4 Department of Obstetrics and Gynecology, Elias Emergency University Hospital, Carol Davila University of Medicine and Pharmacy, 020021 Bucharest, Romania; alexandru.filipescu@umfcd.ro

**Keywords:** endometriosis, chronic pelvic pain, repetitive transcranial magnetic stimulation, neuromodulation, primary motor cortex, pain interference, real-world evidence

## Abstract

Pelvic pain may persist despite guideline-concordant endometriosis care. Evidence for primary motor cortex repetitive transcranial magnetic stimulation (M1-rTMS) in this population is limited. We assessed daily pain and activity interference, treatment delivery and safety after clinically indicated M1-rTMS. A prospective, single-centre observational cohort enrolled women with confirmed endometriosis and chronic pelvic pain. Ten left M1-rTMS sessions were scheduled over two weeks (10 Hz, 80% resting motor threshold, 1500 pulses/session). Daily diaries recorded pelvic pain and activity interference during pre-treatment, treatment, and post-treatment days 1–28 and week 8. Changes were estimated using linear mixed-effects models. Forty-two participants were enrolled and 40 initiated treatment; analyses included 37 for pain and 35 for interference. Model-based marginal mean pain decreased from 5.69 to 4.69 (change, −1.00; 95% CI, −1.46 to −0.55; *p* < 0.001), and interference from 6.03 to 4.63 (change, −1.39; 95% CI, −1.84 to −0.95; *p* < 0.001). Both remained below pre-treatment at week 8. Ten of 34 evaluable participants achieved ≥30% pain reduction and three achieved ≥50%. Of the 400 planned sessions, 386 were delivered; a total of 33/40 participants attended all ten and 38/40 at least eight. Twenty-eight participants reported 47 treatment-emergent adverse events; one was severe, none was serious, and all were resolved. M1-rTMS was feasible to deliver with high protocol fidelity and acceptable tolerability. Modest reductions in pain and activity interference were observed, although clinically important pain relief occurred in a minority; without a sham, usual-care, or untreated comparator, these changes cannot be attributed causally to rTMS. Controlled trials are required to determine efficacy and optimal dosing.

## 1. Introduction

Endometriosis affects roughly one in ten women of reproductive age and is defined by endometrial-like tissues outside the uterus [1]. It commonly causes dysmenorrhoea, dyspareunia, non-menstrual pelvic pain and impaired quality of life. Hormonal treatment and surgery remain the mainstays of disease-directed care, but response varies, symptoms often recur, and some women continue to experience pelvic pain despite adequately attempted treatment [1,2]. Persistent pain can disrupt sleep, work, sexual function and emotional well-being, and further disease-directed treatment may not address the mechanisms that sustain it [2].

Pain severity correlates only weakly with the anatomical extent of the disease [1,3]. Nociceptive input from lesions may combine with pelvic-floor or myofascial dysfunction, visceral cross-sensitisation, altered descending modulation and central amplification, and the relative contribution of these mechanisms differs between patients [2,3]. This may partly explain why pain persists after hormonal or surgical treatment in some women. Psychophysical measures such as remote pressure–pain thresholds can characterise altered pain sensitivity, but neither of these measures nor any single questionnaire can establish central sensitisation or nociplastic pain in isolation [3,4]. Treatments targeting central pain processing are therefore of interest as adjuncts within multidisciplinary care.

Repetitive transcranial magnetic stimulation (rTMS) alters cortical excitability non-invasively. High-frequency stimulation of the primary motor cortex (M1) has produced analgesic effects across several chronic pain conditions, with the strongest evidence in neuropathic pain, and is thought to engage distributed pain-modulatory networks rather than the stimulated cortex alone [5]. This provides a rationale for studying M1-rTMS as an adjunct for persistent endometriosis-associated pain. However, benefits observed in other chronic pain conditions may not necessarily extend to endometriosis.

Endometriosis-specific evidence is limited to a single open-label pilot in which 12 women completed five consecutive sessions of high-frequency neuronavigated M1-rTMS [6]. The pilot collected daily pain ratings for 28 days before treatment and for 28 days beginning with the first rTMS session, and reported a modest reduction in pain alongside improvements in brief pain inventory (BPI) interference and selected quality-of-life domains. However, interference was assessed only at scheduled visits, and the 28-day follow-up period combined the treatment course with the days after its completion. Changes during stimulation and changes after treatment therefore could not be distinguished, and pain and interference were not followed as parallel daily trajectories. The sample of 12 participants also precluded meaningful exploration of clinical heterogeneity, treatment delivery and implementation.

Daily electronic diaries are well suited to this setting because endometriosis-associated pain fluctuates and short recall periods reduce the averaging imposed by longer look-back intervals. Endometriosis-specific daily pain and pain-impact measures have undergone content validation and have shown reliability, convergent validity and responsiveness [7,8]. Recording pain intensity and activity interference separately distinguishes changes in symptom severity from changes in the effect of pain on usual activities. An observational cohort can also address questions that are not the primary focus of an efficacy trial: who enters and completes treatment, how faithfully treatment is delivered, how symptoms change from day to day, and the safety and acceptability of the pathway in routine practice. Combined with ultrasound phenotyping, pressure–pain-threshold testing and session-level treatment records, these trajectories can be interpreted in their clinical context.

We therefore conducted a prospective observational cohort study of clinically indicated M1-rTMS in women with endometriosis-associated chronic pelvic pain. The primary objective was to quantify within-participant change in daily global pelvic pain from pre-treatment to the first 28 days after the last session was delivered; the key secondary objective was the corresponding change in daily activity interference due to pelvic pain. Further objectives covered week-8 outcomes, health-related quality of life, patient-perceived change and the symptom each participant selected as most troublesome, together with pressure–pain thresholds and the implementation, fidelity, safety and acceptability of the pathway itself. The patient-reported outcome framework reflected consensus priorities for endometriosis research [9]. One prespecified exploratory analysis examined whether coexisting adenomyosis modified the observed pain trajectory. We hypothesised that daily pain and interference would be lower after treatment than during pre-treatment.

## 2. Materials and Methods

### 2.1. Study Design, Setting and Participants

This prospective, single-centre observational cohort study with intensive longitudinal assessment was conducted at Elias Emergency University Hospital, a tertiary university referral hospital in Bucharest, Romania, from 16 January to 31 July 2026. Consecutive pathway logging occurred from 16 January to 6 May 2026. Women with a documented clinical indication underwent research screening during the same period, and follow-up ascertainment closed on 31 July 2026. Participants with confirmed endometriosis and chronic pelvic pain were followed from a prospective pre-treatment period through a clinically indicated course of primary motor cortex repetitive transcranial magnetic stimulation (M1-rTMS) and for eight weeks after the last session was delivered. Daily electronic reporting was combined with standardised gynaecological, patient-reported, psychophysical, treatment-delivery and safety assessments. Reporting followed the Strengthening the Reporting of Observational Studies in Epidemiology (STROBE) and Template for Intervention Description and Replication (TIDieR) guidelines [10,11].

Women evaluated within the multidisciplinary endometriosis-pain pathway for possible M1-rTMS were entered consecutively in a minimal pathway log during the prespecified period. The log recorded the date and outcome of the routine clinical decision. Clinical indication was determined independent of the study, and any reason for non-indication was recorded. Women with a documented clinical indication were subsequently assessed against the prespecified research eligibility criteria, and only those meeting all criteria were invited. Research participation did not affect treatment eligibility or scheduling priority. It did not alter stimulation parameters, concomitant care or clinical follow-up. Women who declined research participation remained eligible for treatment through the same clinical pathway.

No randomisation, sham stimulation, investigator-assigned delay or other research-mandated comparator was used. The study was designed to characterise trajectories following clinically indicated rTMS, not to estimate comparative treatment effects. Treatment indication, scheduling, uptake, attendance, protocol deviations and concomitant care arose from routine practice. The diary, questionnaires, study ultrasound and pressure–pain-threshold assessments were collected specifically for research. Women were eligible if they were aged 18 years or older and had not undergone natural or surgical menopause; treatment-induced amenorrhoea and hysterectomy with ovarian preservation were permitted. Endometriosis required confirmation by histology, unequivocal operative documentation, expert transvaginal ultrasonography showing an ovarian endometrioma or deep endometriosis, or magnetic resonance imaging (MRI) showing either finding and interpreted by an experienced radiologist. Isolated superficial peritoneal disease required surgical documentation. Concomitant adenomyosis was permitted, but isolated adenomyosis was insufficient for eligibility [1].

Pelvic pain had to persist or recur for at least six months, occur outside menstrual bleeding rather than being limited to dysmenorrhoea, and average at least 4 on a 0–10 numerical rating scale (NRS) during the preceding seven days at screening [12]. For women with treatment-induced amenorrhoea or previous hysterectomy, eligibility required pain that was not confined to predictable cyclical episodes. The screening NRS was used only to determine whether the prespecified pain threshold was met; it was distinct from the prospective diary and was not used to reconfirm eligibility. Participants also had to have persistent pelvic pain despite at least one guideline-recommended treatment that had been adequately tried. Previous treatment was also considered sufficient for eligibility when it had been discontinued because of adverse effects or contraindications, or declined after appropriate counselling.

Hormonal therapy had to be unchanged for at least 12 weeks before session 1. Scheduled analgesics, centrally acting pain medication and ongoing physical or psychological treatments had to be stable for at least four weeks, with no major change planned before week 4. Rescue medication and medically necessary treatment changes remained permitted throughout, were documented prospectively and did not trigger automatic withdrawal.

Women were excluded for pregnancy or planned conception before follow-up completion; therapeutic endometriosis surgery within six months; a diagnostic or pain-relevant pelvic procedure within 12 weeks; planned pelvic surgery during follow-up; active pelvic infection, suspected malignancy or another urgent gynaecological condition; or another unstable disorder judged to be the predominant pain source and to require priority treatment. Stable coexisting pain conditions and stable depressive or anxiety disorders were recorded rather than treated as grounds for exclusion.

Transcranial magnetic stimulation (TMS) safety was assessed during the routine clinical evaluation and checked again at research screening and before treatment. Women with a contraindication identified during the initial clinical evaluation were not recommended for rTMS. A contraindication identified later precluded research eligibility or treatment initiation, depending on when it was detected. TMS-related exclusions were an incompatible implanted electronic device or intracranial ferromagnetic material, epilepsy or a previous unprovoked seizure, an unacceptable cumulative seizure-risk profile, unstable serious neurological or medical disease, active psychosis, current mania, acute suicidal intent or plan, inability to consent, previous therapeutic M1-rTMS for pelvic pain, or concurrent participation in another non-invasive brain-stimulation protocol. A family history of epilepsy or potentially proconvulsant medication did not exclude participation in isolation; overall risk was assessed according to current safety recommendations [13]. Previous rTMS for another indication was permitted if it had been completed more than six months earlier and was documented.

### 2.2. Gynaecological Assessment and Phenotyping

After enrolment and before planned treatment initiation, all participants underwent a standardised transvaginal ultrasound examination performed by a gynaecologist with dedicated expertise in endometriosis imaging, using a GE HealthCare Voluson E10 ultrasound system equipped with an RIC5-9-D wideband endovaginal volume transducer (GE Healthcare Austria GmbH & Co OG, Zipf, Austria). Examinations included two-dimensional greyscale and colour Doppler imaging, and a three-dimensional uterine volume was acquired for multiplanar assessment of the junctional zone. The examination followed the International Deep Endometriosis Analysis (IDEA) approach and included systematic assessment of the uterus and adnexa, ovarian mobility and soft markers, the pouch-of-Douglas sliding sign, and the anterior, posterior and parametrial deep-endometriosis compartments [14,15]. Where technically feasible, lesion location, dimensions and relationships to adjacent structures were recorded. Standardised still images, cine loops of dynamic assessments and the three-dimensional uterine volume were archived for quality review.

Adenomyosis features were documented according to the revised Morphological Uterus Sonographic Assessment (MUSA) definitions [16]. For this study, definite adenomyosis required at least one direct MUSA feature; examinations showing indirect features only were classified as indeterminate, and those showing neither direct nor indirect features as absent. The exploratory modifier analysis compared definite with absent adenomyosis; indeterminate examinations were reported separately and excluded from that comparison.

The distribution of endometriosis identified on the study ultrasound was recorded using the #Enzian classification [17]. If a compartment could not be assessed reliably, it was recorded as not assessable rather than disease-free and was excluded when calculating the proportion of involvement for that compartment. MRI was not mandated; available clinical MRI findings were recorded separately from the study-ultrasound phenotype. Historical operative and pathology findings, including surgical #Enzian and revised American Society for Reproductive Medicine classifications, were extracted when adequately documented but were neither merged with the current ultrasound phenotype nor used to overwrite it [17,18]. When more than one qualifying source was available, a single primary confirmation source was assigned for descriptive reporting in the following order: histology, operative documentation, expert ultrasonography, and MRI.

A single expert performed all study examinations. A second experienced examiner independently reviewed a random sample of approximately 20% of the archived examinations for completeness and coding consistency. Any discrepancies were resolved by consensus. Imaging variables were treated as primarily descriptive because the anticipated cohort size did not support reliable modelling across multiple anatomical compartments. Definite concomitant adenomyosis was the only prespecified gynaecological candidate modifier of the pain trajectory.

### 2.3. rTMS Treatment, Fidelity and Safety

Treatment was delivered in the hospital neuromodulation unit using a MagVenture A/S MagPro X100 magnetic stimulator connected to a Cool-B65 actively cooled figure-of-eight coil and an external Cooler Unit (MagVenture A/S, Farum, Denmark), with a standard biphasic pulse waveform. The same coil and waveform were used for motor-hotspot localisation, RMT determination and treatment. Participants were seated with the head supported, the right hand relaxed and hearing protection in place. The target was the left M1 hand representation. Surface electromyography was recorded from the right first dorsal interosseous muscle in a belly–tendon montage using a one-channel MEP Monitor and disposable surface electrodes (MagVenture A/S, Farum, Denmark). The motor hotspot was defined as the position producing the largest and most reproducible motor-evoked potentials, with the coil handle approximately 45° posterolateral to the sagittal plane. Resting motor threshold (RMT) was the lowest stimulator intensity eliciting responses of at least 50 microvolts in at least five of ten trials [19]. Neuronavigation was not used; the hotspot and coil orientation were marked on an individually aligned cap to support repositioning. RMT was determined before sessions 1 and 6 and reassessed after a clinically relevant change likely to alter cortical excitability [19,20].

Each session delivered 10 Hz rTMS at 80% of the current RMT in 15 ten-second trains separated by 50 s intervals, for a total of 1500 pulses. Ten sessions were scheduled once daily on five working days per week over two consecutive weeks, for a planned cumulative dose of 15,000 pulses. The stimulation parameters reproduced those used in the previous endometriosis-specific pilot and were consistent with high-frequency M1-rTMS protocols for chronic pain [5,6]. No study-mandated behavioural intervention was paired with stimulation.

Missed sessions were rescheduled when feasible; no more than one session was delivered per calendar day, and missed pulses were not added to later sessions. Temporary pauses and authorised intensity reductions for tolerability were permitted, but response-guided changes in cortical target, frequency, train duration or intertrain interval were not. Planned and delivered exposure were recorded separately, including session dates, intensity, trains, pulses, interruptions, deviations and their reasons.

Each delivered session was assigned to one of four fidelity categories. A session was protocol-concordant when all 1500 pulses were delivered exactly as prescribed. A session was complete with an authorised modification when all 1500 pulses were delivered after a permitted adjustment. Sessions delivering 1200–1499 pulses were classified as incomplete without a major dose deviation. A major deviation was recorded when fewer than 1200 pulses were delivered or when a major treatment parameter was changed without authorisation. Participant-level treatment-delivery outcomes included cumulative dose, number of sessions attended, the proportions attending all 10 sessions and at least 8 sessions, and reasons for missed or incomplete treatment. Procedure duration was measured from coil positioning to the final pulse. Total visit duration was measured from the pre-session assessment to the end of post-session observation.

Before each session, operators reviewed interval medical events, medication or substance changes, and new factors affecting TMS safety. Pregnancy testing was performed when pregnancy could not be excluded clinically. Common pre-existing symptoms were documented before stimulation, and immediate and delayed symptoms were assessed systematically during treatment and through week 8 [21]. Participants were advised to contact the study team for severe, unexpected or persistent symptoms.

A treatment-emergent adverse event (TEAE) was any unfavourable condition first appearing or worsening after the first therapeutic pulse, irrespective of causality, through week 8. Routine fluctuations in diary-recorded pelvic pain remained outcome data unless clinically distinct, temporally associated with treatment or requiring action. Severity was graded as mild, moderate or severe according to functional consequences and required intervention; seriousness followed the International Council for Harmonisation (ICH) E2A criteria [22]. Each TEAE was assessed for its possible relationship to rTMS, considering its timing, known TMS effects and alternative explanations.

Stimulation was paused whenever continuation was considered unsafe, including after a suspected serious adverse event or acute neurological or psychiatric deterioration [13]. If an adverse event led to a change in the session, it was recorded in both the safety and fidelity logs but counted only once in the adverse-event analysis. Safety was summarised per participant and per delivered session, including episodes per 100 sessions; a persistent unchanged event was counted once unless it worsened or required new action.

### 2.4. Outcomes and Follow-Up

The outcome framework covered overall pain, improvement in the most troublesome symptom and health-related quality of life [9]. The primary outcome was daily global pelvic pain, and the key secondary outcome was daily interference of pelvic pain with usual activities, hereafter termed daily activity interference. Other outcomes were analysed separately, and no composite success score was derived. Post-treatment day 1 was defined as the first calendar day after the last session was actually delivered, including when treatment ended early.

Participants completed a study-specific electronic diary once daily throughout pre-treatment and treatment, during post-treatment days 1–28, and during a seven-day period ending on or immediately before the week-8 assessment. Each entry referred to the preceding 24 h, was time-stamped and could not be reconstructed after the daily reporting window had closed. The diary recorded average global pelvic pain and the extent to which pelvic pain interfered with usual activities during the preceding 24 h, each on a 0–10 NRS, with higher scores indicating greater pain or interference. It also recorded menstrual bleeding, dysmenorrhoea when applicable, and rescue medication by drug, dose and number of administrations. Completion of the pain item defined a valid pain day, and completion of the interference item defined a valid interference day; a missing response to one item did not invalidate the other. Completion of the rescue-medication field, including an explicit response of no use, defined a valid medication-reporting day. The study-specific diary was developed through expert review and cognitive interviews with six non-enrolled women, then piloted electronically for seven days [7,8].

The principal pre-treatment window was the 28 days before session 1. Diary recording began after consent, but treatment was not delayed to complete the full window. When fewer than 28 days were available, all recorded pre-treatment observations were retained. Each longitudinal set required at least 14 valid pre-treatment observations and at least one post-treatment observation of the relevant outcome. The primary and key secondary contrasts compared pre-treatment with post-treatment days 1–28. Treatment-phase observations were summarised descriptively, and the week-8 diary was analysed separately. Observations recorded more than 28 days before session 1 were excluded from the principal pre-treatment window.

Responder analyses compared participant-level pre-treatment and post-treatment means and required at least 14 valid pain observations in both windows. Reductions of at least 30% and 50% defined moderately important and substantial improvement, respectively [23]. Participants with insufficient data were not assigned responder status but could still contribute to the longitudinal models.

Health-related quality of life was assessed at baseline and at weeks 4 and 8 using a Romanian-language version of the RAND 36-Item Health Survey 1.0 prepared for this study [24]. Translation followed a forward–backward procedure with cognitive debriefing; the resulting version had not undergone formal psychometric validation [25]. Domain scores were calculated on a 0–100 scale, with higher scores indicating better health [26].

The Patient Global Impression of Change (PGIC) was completed at weeks 4 and 8; much improved or very much improved defined a favourable response [23]. At baseline, each participant selected the endometriosis-associated pain or functional symptom with the greatest impact during the preceding four weeks from persistent pelvic pain, dysmenorrhoea, dyspareunia, dyschezia, dysuria, fatigue or another specified symptom, and rated its impact from 0 to 10. The symptom selected at baseline was reassessed at weeks 4 and 8 and could not be replaced. If the relevant event had not occurred, the symptom was recorded as not assessable rather than scored as zero [9,27,28]. The nine-item Patient Health Questionnaire (PHQ-9) and the seven-item Generalised Anxiety Disorder scale (GAD-7) were administered at baseline for descriptive purposes [29,30]. Expected pain relief was rated from 0 to 10 at baseline, before planned treatment initiation.

Pressure–pain threshold (PPT) was measured within seven days before session 1 and at week 4 using a calibrated digital algometer with a 1 cm^2^ probe. After at least 10 min of rest and one familiarisation trial, three ascending measurements separated by 30 s were obtained at each site at a rate of approximately 50 kPa/s [31]. Sites were the bilateral rectus abdominis midway between the umbilicus and pubic symphysis and the right tibialis anterior 5 cm distal and 1 cm lateral to the tibial tuberosity [32,33,34]. Site order was randomised at baseline and repeated at follow-up. The same examiner and algometer were used at both assessments, and the examiner did not have access to previous PPT values. The bilateral abdominal mean defined local PPT, and the tibialis anterior value defined remote PPT. Current pain, menstrual bleeding or cycle day, analgesic use during the preceding 24 h and assessment time were recorded. PPTs were treated as supportive psychophysical measures.

Baseline assessment was completed after enrolment. For participants who initiated treatment, it occurred within seven days before session 1. Week 4 targeted day 28 after the last session was delivered, with an acceptable window of days 25–35, and week 8 targeted day 56, with an acceptable window of days 49–63. Both visits included RAND-36, PGIC, reassessment of the selected symptom, medication and intercurrent-event review, and safety follow-up; PPT and acceptability were obtained at week 4 only. The additional week-8 diary covered seven consecutive days ending on or immediately before the visit. Patient-reported measures could be completed remotely using the same mode of administration, whereas PPT required attendance. Assessments completed outside these windows were retained in the scheduled-visit analyses, and deviations from the visit windows were reported. Additional treatments initiated before week 8 were recorded, together with their start dates. No maintenance or booster rTMS was scheduled by the study before week 8. Participants remained eligible for follow-up after treatment discontinuation.

### 2.5. Implementation and Acceptability Outcomes

Implementation outcomes described progression through the clinical pathway and treatment delivery under routine-practice conditions. Counts covered pathway entry, clinical indication, research eligibility, invitation, consent and treatment initiation, together with reasons for non-indication, research ineligibility, non-participation or non-initiation. Intervals were calculated from documented clinical indication to research screening, from research screening to the first session, and from clinical indication to the first session overall.

Treatment delivery was characterised by the number and spacing of sessions, attendance at all 10 and at least 8 scheduled sessions, cumulative pulse dose, and the frequency and reasons for missed sessions. Additional measures included authorised modifications and major deviations, procedure and total visit duration, completion of scheduled follow-up assessments, and diary adherence for the pain and activity interference items by study phase. Treatment-course span, defined as the elapsed number of calendar days between the first and final delivered sessions, was summarised separately among participants who attended all 10 scheduled sessions.

Acceptability was assessed at week 4 among treated participants with available responses. Participants rated overall satisfaction from 0 (not at all satisfied) to 10 (completely satisfied) and indicated whether they would choose the same treatment again under similar circumstances (yes, no or unsure); reasons were recorded for negative or uncertain responses.

### 2.6. Data Management and Quality Assurance

Each participant was assigned a unique study identifier, and identifying information was stored separately from the research dataset in access-controlled institutional systems. Data were entered into REDCap (Research Electronic Data Capture, version 15.5.26) with time-stamped diary submissions and prespecified range and logic checks [35].

Clinical-indication and research-eligibility decisions, treatment exposure, primary pain and key secondary activity interference observations, serious adverse events and assessment dates were verified against source records. All data queries were resolved and the planned ultrasound review was completed before final analysis.

### 2.7. Statistical Analysis

Analyses followed a written statistical analysis plan finalised before outcome analyses were run, including prespecified variable-derivation rules. The statistical analysis plan is provided as Supplementary Material S1. Continuous variables were summarised as mean and standard deviation (SD) or median and interquartile range (IQR), and categorical variables as counts and percentages with explicit denominators. Estimates were accompanied by 95% confidence intervals (CIs), and interpretation emphasised magnitude and precision rather than isolated P values.

The pathway-log population supported aggregate clinical-indication and research-recruitment counts. The enrolled cohort comprised all participants who provided consent, and the treated cohort comprised those who received at least one therapeutic session; the latter was used for treatment-exposure and safety analyses. Outcome-specific longitudinal sets required at least 14 valid pre-treatment observations and at least one post-treatment observation of the relevant outcome. Responder analyses required at least 14 valid pain observations in both principal windows. Reasons for exclusion from each analysis set were described, and baseline characteristics of participants included in and excluded from the primary longitudinal set were compared using standardised mean differences (SMDs) rather than significance tests.

The primary analysis estimated within-participant change in daily pelvic pain from pre-treatment to post-treatment days 1–28. This change was interpreted as an observed trajectory rather than a causal treatment effect. Follow-up continued regardless of treatment completion, protocol deviations or clinically necessary changes in concomitant care, and all subsequent available observations were retained.

The primary linear mixed-effects model included pain observations from the pre-treatment and post-treatment windows. Pre-treatment days were indexed from −28 to −1 relative to session 1. Post-treatment days were indexed from 1 to 28 relative to the last delivered session. Fixed effects were phase and phase-specific natural cubic splines of within-phase day with three degrees of freedom. Participant-specific random intercepts and random slopes for phase were fitted with an unstructured covariance matrix, allowing the pre-treatment-to-post-treatment change to vary between participants. Residual serial dependence was represented by a continuous-time first-order autoregressive correlation structure based on actual dates. The primary contrast was the difference between marginal means averaged across the 28 scheduled days in each phase. Treatment-phase observations were not included in the primary or key secondary models and were summarised descriptively as observed daily means. Model diagnostics were examined for both outcomes; the prespecified fixed-effects, random-effects and residual-correlation structures were retained in the final models.

Four sensitivity analyses were specified. The first adjusted for menstrual bleeding. The second was restricted to participants with at least 80% pain-diary completion in both principal windows. The third used the per-protocol set. The fourth excluded participants who began a major new pain-directed intervention before post-treatment day 28. The per-protocol set required at least eight sessions, each delivering at least 1200 pulses at the prescribed target, frequency and train structure, with only authorised modifications and no major unauthorised deviation.

Participant-level pre-treatment and post-treatment means provided a model-independent secondary estimate. Daily outcomes were not singly imputed. Mixed-effects models used all observed data under a missing-at-random assumption, and completion and reasons for missingness were reported by phase and treatment completion.

Daily activity interference was analysed in a separate outcome-specific set using the same model structure, diagnostic procedures, missing-data handling and sensitivity analyses, with adherence calculated from the expected interference ratings. The key secondary contrast was the corresponding difference between 28-day marginal means. Week-8 comparisons were restricted to the corresponding outcome-specific longitudinal set and required at least four valid ratings during the seven-day week-8 diary for the relevant outcome. For these comparisons, a participant-level mean was calculated for the pre-treatment and week-8 periods, and change was estimated as the paired mean difference. Rescue-medication use was expressed for each participant as the proportion of valid medication-reporting days with any rescue-medication use in each principal window. The paired comparison was restricted to the pain-responder-analysis set and required at least 14 valid medication-reporting days in both windows. The number of administrations and drug classes was summarised descriptively.

For dysmenorrhoea, participant-level means were calculated across assessable diary days in the pre-treatment and post-treatment windows among participants with at least one valid dysmenorrhoea rating in both periods.

RAND-36 changes were estimated using a linear mixed-effects model with categorical visit and participant-specific random intercepts. PGIC was summarised as the proportion with a favourable response. Changes in the selected-symptom impact score were estimated as paired mean differences among participants with assessable responses at both relevant visits. Local and remote PPT changes were analysed separately as paired mean differences, with bootstrap confidence intervals used when the distribution of within-participant differences was markedly non-normal. For week-8 diary outcomes, dysmenorrhoea, rescue-medication proportions and selected-symptom impact, 95% confidence intervals for paired mean differences were based on the t distribution. Pain-responder and favourable-PGIC proportions were reported with Wilson 95% confidence intervals, with exact intervals used when counts were sparse. Missing paired observations were not imputed.

Definite versus absent adenomyosis was examined through an interaction with the pre-treatment-to-post-treatment contrast; indeterminate examinations were excluded. This hypothesis-generating analysis was interpreted from the interaction estimate and its confidence interval. Only the primary pain contrast was principal, daily activity interference was the key secondary outcome, and all remaining analyses were supportive or exploratory. No formal adjustment for multiplicity was applied.

Recruitment was calendar-based. Before data collection, approximately 36 participants were expected to contribute to the primary longitudinal analysis. Assuming a standard deviation of 2.0 for within-participant change, this corresponded to a 95% confidence-interval half-width of approximately 0.68 NRS points. The study was not designed for definitive modifier inference.

Analyses used R (version 4.6.0). A second investigator reviewed the variable derivations, the code for the primary and key secondary analyses, and the principal outputs. Two-sided tests used an alpha level of 0.05, with *p* values interpreted alongside confidence intervals and the analysis hierarchy.

### 2.8. Ethics

The study was conducted in accordance with the Declaration of Helsinki and approved by the local Institutional Ethics Committee. All enrolled participants provided written informed consent before research-specific procedures.

## 3. Results

### 3.1. Participant Flow and Analysis Populations

**Figure 1:**
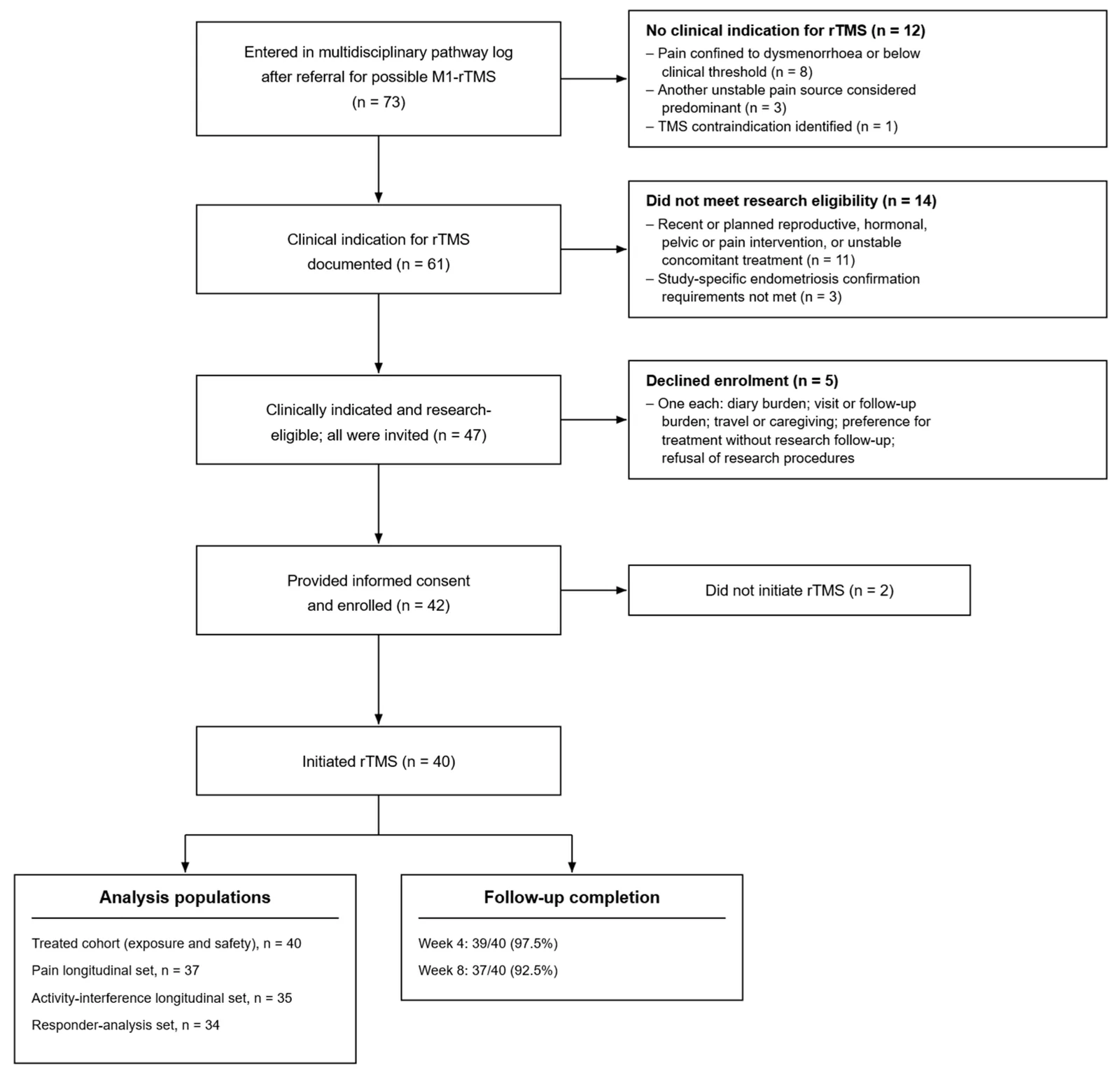
Participant flow through the clinical pathway and study analyses. M1-rTMS, repetitive transcranial magnetic stimulation of the primary motor cortex.

Between 16 January and 6 May 2026, 73 women were entered in the multidisciplinary pathway log after referral for possible M1-rTMS (Figure 1). Twelve did not receive a clinical indication: pain was confined to dysmenorrhoea or below the clinical threshold in eight, another unstable pain source was considered predominant in three, and a TMS contraindication was identified in one. Of the 61 women with a documented clinical indication, 14 did not meet the research eligibility criteria. Eleven had a recent or planned reproductive, hormonal, pelvic or structured pain intervention, or unstable concomitant treatment, and three did not meet the study-specific requirements for endometriosis confirmation. All 47 clinically indicated and research-eligible women were invited, and 42 consented. Each of the five women who did not enrol gave a different reason: diary burden; treatment-visit or follow-up burden; travel or caregiving constraints; a preference for treatment without research follow-up; or refusal of research procedures.

Forty of the 42 enrolled participants initiated rTMS (95.2%). Two enrolled participants did not initiate treatment: one developed an intercurrent medical illness before session 1, and one withdrew her preference for treatment before initiation. Among clinically indicated women, the median interval from the clinical decision to research screening was 12.0 days (IQR, 6.0–17.0; range, 0–21). Among treated participants, the median interval from research screening to the first session was 34.0 days (IQR, 31.8–40.3; range, 20–51), yielding an overall median indication-to-first-session interval of 45.0 days (IQR, 39.8–50.0; range, 30–64). The last participant enrolled on 17 April, the final rTMS session was delivered on 27 May, and the latest week-8 assessment was completed on 28 July. Follow-up ascertainment closed on 31 July.

The pain and activity interference longitudinal sets included 37 and 35 participants, respectively; exclusions beyond the two non-initiators reflected fewer than 14 valid pre-treatment ratings (pain, *n* = 2; interference, *n* = 4) or no post-treatment ratings (*n* = 1). Thirty-four participants met the responder-analysis requirements; three otherwise eligible participants had fewer than 14 valid post-treatment pain ratings. Week-4 and week-8 assessments were completed by 39/40 (97.5%) and 37/40 (92.5%); one completed visit at each time point occurred outside the prespecified window. Among week-8 completers, 35 met the diary requirement for pain and 33 met the requirement for activity interference. The week-4 noncompletion reflected a scheduling conflict. The three week-8 noncompletions reflected inability to establish contact, a participant’s decision to end research follow-up because of time burden, and an intercurrent family or medical circumstance.

### 3.2. Baseline Characteristics and Gynaecological Phenotype

The enrolled cohort had a mean age of 37.6 ± 7.9 years and a mean body mass index (BMI) of 25.4 ± 4.4 kg/m^2^. Median pelvic pain duration was 94 months (IQR, 69.2–120.8), and mean screening pain was 5.7 ± 0.9. Previous therapeutic surgery and stable hormonal therapy were each reported by 33/42 participants (78.6%), scheduled analgesic use by 17/42 (40.5%), and coexisting pain conditions by 18/42 (42.9%) (Table 1). Five enrolled participants were excluded from the primary longitudinal set; standardised differences were interpreted descriptively because of the small excluded group.

The primary qualifying source of endometriosis confirmation was histology in 23 participants, operative documentation in 10, expert ultrasonography in six and MRI in three. Study ultrasonography showed deep endometriosis in 28/42 participants (66.7%) and an ovarian endometrioma in 13/42 (31.0%). Neither deep endometriosis nor an ovarian endometrioma was visualised in 10/42 participants (23.8%) despite qualifying historical confirmation. Adenomyosis was classified as definite in 14/42 (33.3%), indeterminate in 5/42 (11.9%) and absent in 23/42 (54.8%). Detailed #Enzian findings and compartment-specific denominators are presented in Table 2. Nine examinations underwent independent quality review; two coding discrepancies were identified and resolved before analysis.

### 3.3. Treatment Uptake, Exposure, Fidelity, and Data Completeness

The 40 treated participants received 386/400 planned sessions (96.5%), 33/40 (82.5%) attended all 10 scheduled sessions and 38/40 (95.0%) attended at least eight sessions. The median cumulative dose was 15,000 pulses (range, 9000–15,000). Seven participants missed 14 sessions in total, mainly because of intercurrent illness, work or caregiving conflicts, and travel difficulty; one participant discontinued treatment after a severe headache. Among the 33 participants who attended all 10 scheduled sessions, the median elapsed course span was 13.0 days (IQR, 11.0–13.0; range, 11–13).

Of the 386 delivered sessions, 346 (89.6%) were fully protocol-concordant, 36 (9.3%) were complete with an authorised modification, two (0.5%) were incomplete without a major dose deviation, and two (0.5%) involved a major deviation. At participant level, 12/40 (30.0%) had at least one authorised modification and 36/40 (90.0%) met the per-protocol definition. Mean total visit duration was 33.7 ± 3.8 min.

The available pre-treatment diary window had a median duration of 28.0 days (IQR, 27.5–28.0; range, 18–28). Median diary adherence exceeded 85% for both outcomes in every study phase (Table 3). Thirty-one of 37 participants met the ≥80% pain-diary criterion across both principal windows, and 28 of 35 met the corresponding activity interference criterion.

### 3.4. Daily Pelvic Pain and Activity Interference

The modelled pre-treatment pain trajectory was stable, with an estimated change of +0.09 NRS points from day 1 to day 28 (95% CI, −0.22 to 0.39). In the primary analysis (*n* = 37), marginal mean pain decreased from 5.69 during pre-treatment to 4.69 across post-treatment days 1–28, corresponding to a change of −1.00 NRS points (95% CI, −1.46 to −0.55; *p* < 0.001). Participant-level and sensitivity estimates were consistent with the model-based estimate (Table 4).

**Figure 2:**
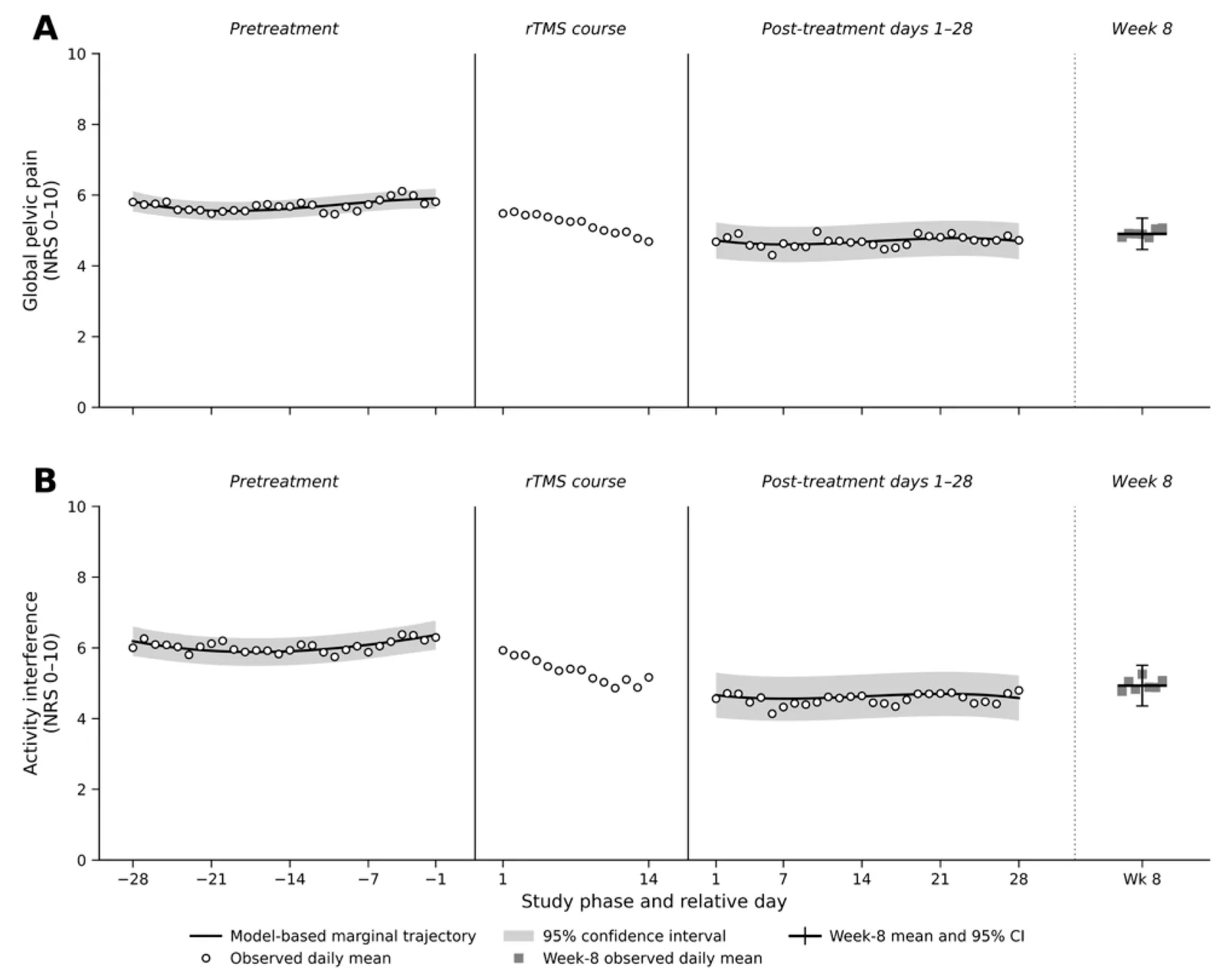
Daily trajectories of (**A**) global pelvic pain and (**B**) activity interference due to pelvic pain. Points represent observed daily means. Lines and shaded bands represent model-based marginal trajectories and 95% confidence intervals during pre-treatment and post-treatment days 1–28. Treatment-phase means are presented descriptively, with daily denominators varying according to available observations. Week-8 means and 95% confidence intervals are shown separately. Pain analyses included 37 participants (week 8, *n* = 35), and interference analyses included 35 participants (week 8, *n* = 33). CI, confidence interval; NRS, numerical rating scale; rTMS, repetitive transcranial magnetic stimulation.

Observed daily mean pain declined during treatment. The modelled post-treatment trajectory remained relatively stable across days 1–28 (Figure 2). Among the 34 participants with sufficient data, 10 achieved a reduction of at least 30% (29.4%; 95% CI, 16.8–46.2), and three achieved a reduction of at least 50% (8.8%; 95% CI, 3.0–23.0). At week 8, mean pain remained 0.80 points below pre-treatment (95% CI, −1.17 to −0.42; *n* = 35).

The modelled pre-treatment trajectory for activity interference was stable, with an estimated change of +0.12 points from day 1 to day 28 (95% CI, −0.22 to 0.46). In the key secondary analysis (*n* = 35), marginal mean interference decreased from 6.03 during pre-treatment to 4.63 across post-treatment days 1–28, corresponding to a change of −1.39 NRS points (95% CI, −1.84 to −0.95; *p* < 0.001). Participant-level and sensitivity estimates were consistent with the corresponding model-based estimate (Table 4). Observed daily means suggested a gradual decline in activity interference during the rTMS course. The modelled post-treatment trajectory remained relatively stable across days 1–28. At week 8, mean interference remained 1.05 points below pre-treatment (95% CI, −1.41 to −0.69; *n* = 33).

### 3.5. Supportive Patient-Reported Outcomes and Rescue Medication

At week 4, 18/39 participants reported being much improved or very much improved on PGIC, compared with 15/37 at week 8. The impact score for the participant-selected symptom decreased by approximately one point at week 4 and 0.7 points at week 8. Dysmenorrhoea decreased by approximately 1.1 points among 25 evaluable participants, and the proportion of diary days with rescue-medication use decreased by 9.7 percentage points among 34 participants (Table 5).

RAND-36 improvements at week 4 were observed in bodily pain, physical-role limitation, social functioning, energy/fatigue and physical functioning. Week-8 estimates were smaller for most domains, while improvement in social functioning was maintained. Three participants began a major new pain-directed intervention before post-treatment day 28. Their observations were retained in the main analysis and excluded from the corresponding sensitivity analysis.

Paired PPT data were available for 32 participants. Local abdominal PPT increased by 28.4 kPa (95% CI, 19.4–37.4), whereas remote tibialis-anterior PPT changed by 10.7 kPa (95% CI, −6.4 to 27.9).

The exploratory adenomyosis analysis included 32 participants: 21 with absent and 11 with definite adenomyosis. Five participants with indeterminate examinations were excluded. The interaction estimate was +0.56 NRS points (95% CI, −0.48 to 1.59).

### 3.6. Safety and Acceptability

Twenty-eight of 40 treated participants reported at least one TEAE (70.0%; 95% CI, 54.6–81.9). Across 386 delivered sessions, 47 episodes were recorded, corresponding to 12.2 episodes per 100 sessions (95% CI, 8.9–16.2). Of these, 29 were mild, 17 were moderate and one was severe. Headache, fatigue and scalp discomfort were the most frequently reported events. One severe, non-serious headache was resolved the following day but led to treatment discontinuation. All events were resolved, and none met the seriousness criteria (Table 6). Negative or uncertain responses most commonly reflected limited perceived benefit, transient headache or scalp discomfort, and travel or time burden.

## 4. Discussion

In this prospective real-world cohort, a clinically indicated ten-session course of left M1-rTMS was followed by a modest reduction in daily pelvic pain and a numerically greater reduction in daily activity interference during the first 28 days after treatment. The pain estimate was consistent across sensitivity analyses, and both outcomes remained below pre-treatment at approximately eight weeks, with a numerically smaller pain estimate at week 8. Approximately three in ten evaluable participants achieved at least a 30% pain reduction, and fewer than one in ten achieved at least 50%. Treatment uptake, completion, fidelity and diary adherence were high, and acceptability was favourable. Adverse events were common under active surveillance but predominantly mild or moderate; all were resolved, and none was serious.

The only previous endometriosis-specific study was an open-label pilot of five neuronavigated sessions in 12 participants [6]. It reported an approximately one-point reduction in BPI pain intensity and larger reductions in BPI interference, but its 28-day observation period began with the first treatment session and interference was assessed only at scheduled visits. Our cohort adds a larger and more heterogeneous clinical sample, ten-session delivery, and parallel daily tracking of pain and activity interference. Differences in treatment dose, outcome instruments, follow-up timing, and neuronavigation preclude direct comparison of effect magnitude.

The average reduction should be distinguished from individual clinical response. Twenty-nine percent of the evaluable participants achieved at least a 30% pain reduction, and 9% achieved at least 50% [23]. A subgroup therefore experienced clinically important relief, but most participants had smaller changes or no clinically meaningful improvement. This heterogeneity is plausible in endometriosis-associated chronic pelvic pain, in which peripheral nociceptive input, myofascial dysfunction, visceral cross-sensitisation, altered pain modulation and psychosocial factors may contribute in varying proportions [2,3,4]. A transition from persistent visceral nociception to increasingly central neuroplastic mechanisms has also been described in chronic pancreatitis [36].

The numerically greater reduction in daily activity interference may be clinically relevant because pain intensity and interference with usual activities are related but distinct. Reduced rescue-medication use, lower impact of the selected most troublesome symptom, and improvements in selected RAND-36 domains were directionally consistent with reduced symptom burden. The higher PGIC response may reflect participants integrating several dimensions of change rather than pain intensity alone, although PGIC remains vulnerable to expectation and recall bias in an unblinded cohort.

Observed daily means suggested a gradual decline during treatment, while the modelled post-treatment trajectories were relatively stable across days 1–28 (Figure 2). The modelled pre-treatment trajectories were essentially flat for both pain and activity interference, arguing against a downward symptom trend already under way before rTMS initiation. At week 8, mean pain remained below pre-treatment, although the estimated difference was numerically smaller than during post-treatment days 1–28. Pre-treatment stability does not, however, rule out other explanations. Selection based on a screening threshold may introduce regression towards the mean. Expectation and other non-specific effects, natural symptom fluctuation, menstrual and non-menstrual influences, and concomitant care may also have contributed. Menstrual status was heterogeneous across the cohort. Although daily menstrual bleeding was prospectively recorded and included in a prespecified sensitivity analysis, with estimates essentially unchanged from the primary analyses, this does not fully account for differences in cycle phase or ovarian hormonal activity, which may have contributed to individual pain trajectories.

Ninety-five percent of the enrolled participants initiated treatment, 83% attended all 10 scheduled sessions, 95% attended at least eight sessions, and 97% of planned sessions were delivered. Major deviations were rare, visits averaged approximately half an hour, and diary completion exceeded 85% in every principal phase. The median indication-to-treatment interval was 45 days, and among participants attending all 10 scheduled sessions, the course spanned a median of 13 elapsed days. These implementation estimates derive from a single tertiary centre with dedicated study support and may not generalise to settings with different staffing, capacity, travel burden, or costs.

No serious adverse events occurred, and headache, fatigue and scalp discomfort were the predominant TEAEs, consistent with established tolerability patterns for high-frequency M1-rTMS [13]. The high participant-level TEAE proportion should be interpreted in the context of the broad treatment-emergent definition and structured assessment before and after each session. One severe but non-serious headache led to treatment discontinuation. Satisfaction remained favourable, and approximately three quarters of the participants would choose the treatment again.

Local abdominal PPT increased, whereas remote PPT changed little. Repeated assessment may have introduced familiarity or habituation effects, and the PPT findings should therefore be interpreted as supportive psychophysical observations rather than as evidence of a change in central sensitisation or normalisation of widespread pain processing [3,4,33]. Definite adenomyosis was associated with a numerically smaller pain reduction, but the interaction estimate was imprecise and provided no basis for clinical selection.

Evidence for non-invasive brain stimulation in chronic pelvic pain remains limited and methodologically heterogeneous [37]. Small studies in bladder pain syndrome and endometriosis have reported benefits with M1-rTMS or transcranial direct-current stimulation, although their populations and protocols differ [38,39]. A sham-controlled factorial trial comparing active with sham M1-rTMS and five with ten sessions in endometriosis is registered [40]. The present cohort addresses complementary questions concerning treatment uptake, delivery fidelity, daily trajectories, safety and integration within a clinical pathway.

The study had several strengths. Pathway logging was consecutive and covered a predefined period, while routine clinical indication was kept explicitly separate from research eligibility and invitation. Pain and activity interference were assessed daily and in parallel, treatment delivery was documented session by session, and gynaecological phenotyping was standardised. Prespecified longitudinal models, participant-level estimates and sensitivity analyses provided complementary assessments of the observed trajectories.

The principal limitation is the absence of a concurrent sham, usual-care, or untreated comparator, which prevents causal attribution of the observed changes. Because participants were unblinded, subjective outcomes—particularly PGIC and acceptability—may have been influenced by expectation. The single-centre cohort was modest in size, five enrolled participants did not enter the primary longitudinal analysis because of insufficient diary data, and follow-up extended only to approximately eight weeks. The key secondary activity interference item had undergone cognitive testing but not formal psychometric validation. PPT was assessed only at baseline and week 4 and should be interpreted as a supportive psychophysical measure. Modifier analyses and supportive outcomes should therefore be considered exploratory.

These data support the feasibility of delivering M1-rTMS as an adjunct within a specialised endometriosis-pain pathway, but they do not establish efficacy beyond non-specific or time-related effects. Any clinical use should account for the ten-visit burden and the minority rate of clinically important pain response. Future research should prioritise randomised, sham-controlled multicentre trials, with a usual-care comparator where feasible, together with direct dose comparison, longer follow-up, maintenance strategies, and validated daily activity interference measures. Such trials should incorporate participant blinding and assess blinding success. Larger datasets may then support robust investigation of treatment heterogeneity.

## 5. Conclusions

In conclusion, a clinically indicated ten-session course of M1-rTMS was feasible to deliver with high protocol fidelity and no serious adverse events in this single-centre endometriosis-pain pathway. It was followed by a modest reduction in daily pelvic pain and a numerically greater reduction in daily activity interference; both outcomes remained below pre-treatment at week 8, while clinically important pain relief occurred in a minority. Controlled trials are required to determine efficacy and optimal dosing.

## Figures and Tables

**Table 1:** Baseline characteristics of the enrolled cohort according to inclusion in the primary pain longitudinal analysis.

Characteristic	Enrolled Cohort (*n* = 42)	Primary Longitudinal Set (*n* = 37)	Excluded from Primary Set (*n* = 5)	SMD
Age, years	37.6 ± 7.9	37.5 ± 7.8	38.4 ± 9.5	−0.10
BMI, kg/m^2^	25.4 ± 4.4	25.5 ± 4.4	24.3 ± 5.2	0.26
Pelvic pain duration, months	94.0 (69.2–120.8)	97.0 (71.0–121.0)	62.0 (48.0–99.0)	0.62
Screening pain NRS	5.7 ± 0.9	5.8 ± 0.9	5.5 ± 1.0	0.31
Diagnostic delay, years	7.1 (4.5–9.6)	7.0 (4.5–9.4)	8.5 (4.0–9.7)	0.13
Menstrual status: cycling	28 (66.7)	23 (62.2)	5 (100.0)	−1.10
Treatment-induced amenorrhoea	11 (26.2)	11 (29.7)	0 (0.0)	0.92
Hysterectomy with ovarian preservation	3 (7.1)	3 (8.1)	0 (0.0)	0.42
Previous therapeutic surgery	33 (78.6)	29 (78.4)	4 (80.0)	−0.04
Stable hormonal therapy	33 (78.6)	29 (78.4)	4 (80.0)	−0.04
Scheduled analgesic	17 (40.5)	16 (43.2)	1 (20.0)	0.52
Centrally acting pain medication	13 (31.0)	9 (24.3)	4 (80.0)	−1.34
Coexisting pain condition	18 (42.9)	17 (45.9)	1 (20.0)	0.57
Stable depressive/anxiety disorder	20 (47.6)	18 (48.6)	2 (40.0)	0.17
PHQ-9 total	6.0 ± 3.9	5.9 ± 3.8	6.8 ± 4.7	−0.22
GAD-7 total	5.1 ± 3.4	5.0 ± 3.3	6.2 ± 4.8	−0.30
Expectation of pain reduction, NRS	6.4 ± 1.5	6.4 ± 1.6	6.4 ± 0.7	−0.03

Values are mean ± SD, median (IQR), or *n* (%). SMDs compare the primary longitudinal set with enrolled participants excluded from that set; no significance tests were performed. Abbreviations: BMI, body mass index; GAD-7, seven-item Generalised Anxiety Disorder scale; IQR, interquartile range; NRS, numerical rating scale; PHQ-9, nine-item Patient Health Questionnaire; SD, standard deviation; SMD, standardised mean difference.

**Table 2:** Endometriosis confirmation and study-ultrasound phenotype.

Gynaecological Characteristic	Result
Primary qualifying confirmation source: histology	23/42 (54.8%)
Primary qualifying confirmation source: operative documentation	10/42 (23.8%)
Primary qualifying confirmation source: expert transvaginal ultrasonography	6/42 (14.3%)
Primary qualifying confirmation source: MRI	3/42 (7.1%)
Deep endometriosis on study ultrasound	28/42 (66.7%)
Ovarian endometrioma on study ultrasound	13/42 (31.0%)
Largest endometrioma diameter, mm	37.0 (24.0–42.0); *n* = 13
Neither deep endometriosis nor ovarian endometrioma visualised	10/42 (23.8%)
Adenomyosis: definite	14/42 (33.3%)
Adenomyosis: indeterminate	5/42 (11.9%)
Adenomyosis: absent	23/42 (54.8%)
Negative pouch-of-Douglas sliding sign	6/42 (14.3%)
Soft markers present	18/42 (42.9%)
Restricted left ovarian mobility	10/42 (23.8%)
Restricted right ovarian mobility	8/42 (19.0%)
#Enzian A involvement	13/42 (31.0%)
#Enzian B left involvement	10/39 (25.6%)
#Enzian B right involvement	5/41 (12.2%)
#Enzian C involvement	3/40 (7.5%)
#Enzian O involvement (any ovary)	13/42 (31.0%)
#Enzian T left/right involvement	1/42 (2.4%)/2/42 (4.8%)
#Enzian FB/FI/FU involvement	3/42 (7.1%)/4/39 (10.3%)/1/38 (2.6%)
Independent ultrasound review	9/42 (21.4%)

Endometrioma diameter was summarised among participants with an endometrioma (*n* = 13). Compartments coded as not assessable were excluded from compartment-specific denominators. The #Enzian P compartment was not assigned from study ultrasonography because superficial peritoneal endometriosis cannot be reliably mapped sonographically. Historical operative and MRI findings, including operative #Enzian P findings, were retained separately and were not merged with the current study-ultrasound phenotype. Absence of deep endometriosis or an endometrioma on study ultrasonography did not exclude superficial peritoneal or previously treated disease. MRI, magnetic resonance imaging.

**Table 3:** Clinical pathway, treatment uptake, exposure, fidelity, follow-up completion, and diary adherence.

Implementation/Fidelity Outcome	Result
Clinical pathway and research recruitment	
Entered in pathway log	73
Clinical indication documented	61/73 (83.6%)
Study-eligible among clinically indicated	47/61 (77.0%)
Invited among study-eligible	47/47 (100.0%)
Consented among invited	42/47 (89.4%)
Treatment initiated among enrolled	40/42 (95.2%)
Clinical-indication-to-research-screening interval, days	12.0 (6.0–17.0); range: 0–21
Research-screening-to-first-session interval, days	34.0 (31.8–40.3); range: 20–51
Clinical-indication-to-first-session interval, days	45.0 (39.8–50.0); range: 30–64
Treatment exposure and fidelity	
Delivered sessions	386/400 planned (96.5%)
Attended all 10 scheduled sessions	33/40 (82.5%)
Attended ≥8 scheduled sessions	38/40 (95.0%)
Sessions attended per participant	Median: 10; range: 6–10
Course span among participants attending all 10 sessions, elapsed days	13.0 (11.0–13.0); range: 11–13
Cumulative pulse dose	Median: 15,000; range: 9,000–15,000
Complete protocol-concordant sessions	346/386 (89.6%)
Complete sessions with authorised modification	36/386 (9.3%)
Incomplete sessions without major dose deviation	2/386 (0.5%)
Sessions with major deviation	2/386 (0.5%)
Participant-level fidelity and visit duration	
Participants with ≥1 authorised modification	12/40 (30.0%)
Participants meeting the per-protocol definition	36/40 (90.0%)
Procedure duration, min	20.2 ± 2.0
Total visit duration, min	33.7 ± 3.8
Follow-up and diary completeness	
Available pre-treatment diary window, days	28.0 (27.5–28.0); range, 18–28
Week-4 assessment completed	39/40 (97.5%); 38/39 completed visits within window
Week-8 assessment completed	37/40 (92.5%); 36/37 completed visits within window
Pain-diary adherence: pre-treatment	90.6% (85.7–95.8)
Pain-diary adherence: treatment	91.7% (85.1–100.0)
Pain-diary adherence: post-treatment	92.9% (82.1–96.4)
Pain-diary adherence: week 8	85.7% (71.4–100.0)
Interference-item adherence: pre-treatment	89.3% (82.5–95.8)
Interference-item adherence: treatment	91.7% (85.1–100.0)
Interference-item adherence: post-treatment	89.3% (82.1–96.4)
Interference-item adherence: week 8	85.7% (71.4–100.0)

Values are *n* (%), mean ± standard deviation, or median (interquartile range), as applicable. Adherence values are participant-level medians (IQR). Pathway measures use sequential denominators. Session-level percentages use all delivered sessions unless otherwise specified. The available pre-treatment diary window was calculated from diary activation to the first rTMS session and capped at 28 calendar days. Treatment-course span was the elapsed number of calendar days between the first and final delivered sessions.

**Table 4:** Primary and key secondary outcome analyses and prespecified sensitivity analyses.

Outcome and Analysis	*n*	Estimated Mean Change, NRS Points	95% CI	*p* Value
Daily pelvic pain				
Primary longitudinal model	37	−1.00	−1.46 to −0.55	<0.001
Participant-level pre-treatment and post-treatment means	37	−1.00	−1.46 to −0.55	—
Adjusted for daily menstrual bleeding	37	−1.01	−1.46 to −0.55	—
Restricted to ≥80% pain-diary completion in both windows	31	−1.01	−1.51 to −0.51	—
Per-protocol analysis—pain	34	−1.07	−1.55 to −0.60	—
Excluding major new intervention—pain	34	−0.93	−1.38 to −0.48	—
Daily activity interference due to pelvic pain				
Key secondary longitudinal model	35	−1.39	−1.84 to −0.95	<0.001
Participant-level pre-treatment and post-treatment means	35	−1.40	−1.85 to −0.94	—
Adjusted for daily menstrual bleeding	35	−1.40	−1.85 to −0.95	—
Restricted to ≥80% activity-interference-item completion in both windows	28	−1.65	−2.16 to −1.15	—
Per-protocol analysis	32	−1.43	−1.92 to −0.95	—
Excluding participants who initiated a major new pain-directed intervention before post-treatment day 28	32	−1.35	−1.81 to −0.89	—

Changes represent post-treatment days 1–28 minus the pre-treatment period; negative values indicate improvement. Primary and key secondary estimates compare model-based marginal means averaged across the 28 days of each phase. Sensitivity analyses were interpreted from effect estimates and 95% confidence intervals without separate inferential emphasis. CI, confidence interval; NRS, numerical rating scale.

**Table 5:** Supportive clinical, patient-reported, and psychophysical outcomes.

Supportive Outcome	Time/Analysis	*n*	Baseline	Follow-Up	Estimate (95% CI)
Daily pelvic pain	Week-8 diary vs. pre-treatment	35	5.70	4.90	−0.80 NRS (−1.17 to −0.42)
Daily activity interference due to pelvic pain	Week-8 diary vs. pre-treatment	33	5.98	4.93	−1.05 NRS (−1.41 to −0.69)
Pain responder ≥30%	Post-treatment days 1–28	34	—	—	10/34 (29.4%; 95% CI, 16.8–46.2)
Pain responder ≥50%	Post-treatment days 1–28	34	—	—	3/34 (8.8%; 95% CI, 3.0–23.0)
PGIC much/very much improved	Week 4	39	—	—	18/39 (46.2%; 95% CI, 31.6–61.4)
PGIC much/very much improved	Week 8	37	—	—	15/37 (40.5%; 95% CI, 26.3–56.5)
Most troublesome symptom impact	Week 4 vs. baseline	38	7.31	6.29	−1.01 NRS (−1.43 to −0.60)
Most troublesome symptom impact	Week 8 vs. baseline	36	7.26	6.51	−0.74 NRS (−1.07 to −0.42)
Dysmenorrhoea	Post-treatment vs. pre-treatment	25	7.10	6.03	−1.07 NRS (−1.50 to −0.64)
Diary days with rescue medication	Post-treatment vs. pre-treatment	34	52.8%	43.1%	−9.7 percentage points (−17.4 to −2.1)
RAND-36 physical functioning	Week 4/week 8	39/37	75.5/74.3	79.9/76.6	+4.57 (1.65 to 7.49)/+2.07 (−0.91 to 5.04)
RAND-36 role limitations—physical	Week 4/week 8	39/37	28.2/29.7	36.8/30.4	+8.53 (3.02 to 14.04)/+0.72 (−4.90 to 6.35)
RAND-36 role limitations—emotional	Week 4/week 8	39/37	35.9/36.9	40.2/40.5	+3.92 (−3.05 to 10.88)/+4.05 (−3.06 to 11.15)
RAND-36 energy/fatigue	Week 4/week 8	39/37	31.4/32.0	35.8/33.4	+4.19 (1.97 to 6.42)/+1.11 (−1.17 to 3.38)
RAND-36 emotional well-Being	Week 4/week 8	39/37	52.8/52.5	53.4/52.8	+0.70 (−1.05 to 2.46)/+0.17 (−1.62 to 1.96)
RAND-36 social functioning	Week 4/week 8	39/37	48.4/46.6	53.8/50.7	+5.47 (2.42 to 8.51)/+3.90 (0.80 to 7.01)
RAND-36 bodily pain	Week 4/week 8	39/36	31.3/31.5	38.5/33.5	+7.11 (4.15 to 10.07)/+2.00 (−1.05 to 5.04)
RAND-36 general health	Week 4/week 8	39/37	53.7/53.7	54.6/53.3	+0.89 (−1.12 to 2.89)/−0.49 (−2.53 to 1.55)
Local abdominal PPT	Week 4 vs. baseline	32	260.0 kPa	288.4 kPa	+28.4 kPa (19.4 to 37.4)
Remote tibialis-anterior PPT	Week 4 vs. baseline	32	526.1 kPa	536.8 kPa	+10.7 kPa (−6.4 to 27.9)

Baseline and follow-up values are observed means; RAND-36 changes are model-based, and positive values indicate better health status. Unless otherwise specified, continuous change estimates are paired mean differences with 95% confidence intervals based on the t distribution. PGIC and responder estimates are proportions with 95% confidence intervals. All outcomes in this table were supportive and were not adjusted for multiplicity. CI, confidence interval; kPa, kilopascal; NRS, numerical rating scale; PGIC, Patient Global Impression of Change; PPT, pressure–pain threshold; RAND-36, RAND 36-Item Health Survey 1.0.

**Table 6:** Treatment-emergent adverse events and acceptability.

Safety/Acceptability Outcome	Result
Participants with ≥1 TEAE	28/40 (70.0%; 95% CI, 54.6–81.9)
TEAE episodes	47; 12.2 per 100 sessions (95% CI, 8.9–16.2)
Mild/moderate/severe episodes	29/17/1
Headache	15 participants (37.5%)
Fatigue	12 participants (30.0%)
Scalp discomfort	9 participants (22.5%)
Neck discomfort	4 participants (10.0%)
Dizziness	3 participants (7.5%)
Nausea	3 participants (7.5%)
Transient facial twitching	1 participant (2.5%)
Serious adverse events	0
Events resolved	47/47 (100%)
Permanent treatment discontinuation due to TEAE	1/40 (2.5%)
Satisfaction score at week 4 (*n* = 39)	7.2 ± 1.8
Would choose the same treatment again	30/39 (76.9%)
Unsure/would not choose again	6/39 (15.4%)/3/39 (7.7%)

Participants could experience more than one TEAE, and adverse-event categories were not mutually exclusive. Participant-level safety percentages used the treated-cohort denominator (*n* = 40), whereas the session-level event rate used 386 delivered sessions. Week-4 acceptability data were available for 39 participants. CI, confidence interval; TEAE, treatment-emergent adverse event.

## Data Availability

The original contributions presented in this study are included in the article. Further inquiries can be directed to the corresponding authors.

## Supplementary Materials

The following supporting information can be downloaded at https://www.mdpi.com/article/10.3390/life16101686/s1, Supplementary Material S1: Statistical analysis plan.

## Abbreviations

The following abbreviations are used in this manuscript:

BMI	Body mass index
BPI	Brief pain inventory
CI	Confidence interval
GAD-7	Seven-item Generalised Anxiety Disorder scale
ICH	International Council for Harmonisation
IDEA	International Deep Endometriosis Analysis
IQR	Interquartile range
M1	Primary motor cortex
MRI	Magnetic resonance imaging
MUSA	Morphological Uterus Sonographic Assessment
NRS	Numerical rating scale
PGIC	Patient Global Impression of Change
PHQ-9	Nine-item Patient Health Questionnaire
PPT	Pressure–pain threshold
RAND-36	RAND 36-Item Health Survey 1.0
RMT	Resting motor threshold
rTMS	Repetitive transcranial magnetic stimulation
SD	Standard deviation
SMD	Standardised mean difference
STROBE	Strengthening the Reporting of Observational Studies in Epidemiology
TEAE	Treatment-emergent adverse event
TIDieR	Template for Intervention Description and Replication
TMS	Transcranial magnetic stimulation
