# Supplementary material for: Daily Pain and Activity Interference Trajectories Following Primary Motor Cortex rTMS for Endometriosis-Associated Chronic Pelvic Pain: A Prospective Real-World Cohort Study
Source: Life (Basel). 2026 Oct 9;16(10):1686. doi: 10.3390/life16101686 (PMC13705698; doi:10.3390/life16101686)
Supplement: Supplementary file 1 [file life-16-01686-s001.zip › life-4534990-supplementary.pdf]

Statistical Analysis Plan

Prospective observational cohort of clinically indicated M1-rTMS for endometriosis-associated chronic pelvic pain

1. Study framework and objectives

The study is a prospective, single-centre observational cohort embedded in a multidisciplinary endometriosis-pain pathway. Clinical indication for repetitive transcranial magnetic stimulation of the primary motor cortex (M1-rTMS) will be determined independently of research participation. The study includes no randomisation, sham stimulation, investigator-assigned delay or other research-mandated comparator. The analyses will therefore characterise symptom trajectories, treatment delivery and safety following clinically indicated M1-rTMS rather than estimate a comparative or causal treatment effect.

The primary objective is to quantify within-participant change in daily global pelvic pain from the pretreatment period to the first 28 days after the last session delivered. The key secondary objective is the corresponding change in daily activity interference due to pelvic pain. The working hypothesis is that daily pain and activity interference will be lower after treatment than during pretreatment. Further analyses will cover week-8 outcomes, clinically important pain response, health-related quality of life, patient-perceived change, the participant-selected most troublesome symptom, pressure-pain thresholds, rescue-medication use, implementation, treatment fidelity, safety and acceptability. One exploratory analysis will examine whether definite concomitant adenomyosis modifies the observed pain trajectory.

2. Study design and timing

The planned study period is 16 January to 31 July 2026. Consecutive pathway logging will occur from 16 January to 6 May 2026. Women with a documented clinical indication will undergo research screening during the same period. Follow-up ascertainment will close on 31 July 2026. Final outcome analyses will be undertaken after follow-up closure and completion of data cleaning and quality checks.

Ten M1-rTMS sessions are scheduled once daily on five working days per week over two consecutive weeks. Each session will use left M1 stimulation at 10 Hz and 80% of the current resting motor threshold, delivered in 15 ten-second trains separated by 50-second intervals, for 1,500 pulses per session. Missed sessions may be rescheduled when feasible, but no more than one session will be delivered on a calendar day. Follow-up will continue after early treatment discontinuation or protocol deviations.

2.1. Study size and precision

Recruitment will be calendar-based. Approximately 36 participants are expected to contribute to the primary pain longitudinal analysis. Assuming a standard deviation of 2.0 NRS points for within-participant change, this would correspond to an approximate two-sided 95% confidence-interval half-width of 0.68 NRS points. The calculation describes the anticipated precision of the primary estimate rather than a hypothesis-testing sample size.

The study is not designed for definitive inference on treatment-effect modifiers. The adenomyosis interaction is therefore exploratory and will be interpreted from its estimate and confidence interval.

3. Outcome hierarchy and assessment windows

The primary outcome is daily global pelvic pain recorded on a 0–10 numerical rating scale (NRS), with higher scores indicating greater pain. The key secondary outcome is daily activity interference due to pelvic pain, recorded on a separate 0–10 NRS. Each daily entry refers to the preceding 24 hours. Completion of the pain item defines a valid pain day and completion of the interference item defines a valid interference day; missingness of one item does not invalidate the other. Other outcomes will be analysed separately.

**Window**	**Definition**	**Use in analysis**
Pretreatment	Days −28 to −1 relative to session 1. If fewer than 28 days are available after consent and diary activation, all available observations within this window are retained.	Primary and key secondary longitudinal models; participant-level phase means; responder and supportive paired analyses as specified below.
Treatment	From session 1 through the last delivered session.	Descriptive daily means only; not included in the primary or key secondary longitudinal models.
Post-treatment	Days 1–28 after the last session actually delivered. Day 1 is the first calendar day after that session, including when treatment ends early.	Primary and key secondary longitudinal models; participant-level phase means; responder and supportive paired analyses.
Week 8 diary	Seven consecutive days ending on or immediately before the week-8 assessment.	Separate paired analysis; at least four valid ratings are required for the relevant outcome.

Baseline assessment is planned after enrolment and, for participants who initiate treatment, within seven days before session 1. The week-4 visit targets day 28 after the last delivered session, with an acceptable window of days 25–35. The week-8 visit targets day 56, with an acceptable window of days 49–63. Completed assessments outside these windows will be retained and reported as protocol deviations rather than discarded.

4. Analysis populations

**Population**	**Definition**
Pathway-log population	All women entered consecutively in the multidisciplinary clinical pathway log during the prespecified logging period.

**Population**	**Definition**
Enrolled cohort	All participants who provide written informed consent.
Treated cohort / safety set	Enrolled participants who receive at least one therapeutic M1-rTMS session.
Pain longitudinal set	Treated participants with at least 14 valid pretreatment pain ratings and at least one valid post-treatment pain rating.
Activity-interference longitudinal set	Treated participants with at least 14 valid pretreatment interference ratings and at least one valid post-treatment interference rating.
Pain responder set	Treated participants with at least 14 valid pain ratings in both the pretreatment and post-treatment principal windows.
Per-protocol set	Participants attending at least eight sessions, each delivering at least 1,200 pulses at the prescribed target, frequency and train structure, with only authorised modifications and no major unauthorised deviation.
Week-8 outcome sets	The corresponding pain or interference longitudinal set, additionally requiring at least four valid ratings during the seven-day week-8 diary for that outcome.
Rescue-medication paired set	Pain responder set with at least 14 valid medication-reporting days in each principal window.
Paired supportive sets	Participants with the required paired assessable observations for selected-symptom impact, dysmenorrhoea or PPT, as defined in Section 9.
RAND-36 analysis set	Treated participants with at least one valid domain score at baseline, week 4 or week 8; all available valid scores for the domain concerned will contribute to the mixed-effects model.

5. Derived variables and coding rules

5.1 Diary completeness and participant-level means

Outcome-specific diary adherence will be calculated as the number of valid ratings divided by the number of expected days in the relevant phase. For pretreatment, the denominator is the number of calendar days available from diary activation to session 1, capped at 28 days. For treatment, it is the number of calendar days from session 1 through the last delivered session; for post-treatment, 28 days; and for the week-8 diary, seven days. The ≥80% adherence sensitivity analysis requires the threshold to be met in both principal windows for the outcome concerned.

Participant-level phase means are arithmetic means of all valid outcome ratings within the defined phase. No value will be inserted for a missing daily rating. Observations more than 28 days before session 1 and observations outside the first 28 post-treatment days do not contribute to the principal phase means.

5.2 Pain response

For participants in the pain responder set, percentage pain reduction will be calculated as 100 × (pretreatment mean − post-treatment mean) / pretreatment mean. Reductions of at least 30% and at least 50% define moderately important and substantial improvement, respectively. A participant who does not meet the minimum diary requirement will not be assigned responder status.

5.3 Rescue medication and dysmenorrhoea

A medication-reporting day is valid when the rescue-medication field is completed, including an explicit report of no use. For each participant, rescue-medication use will be expressed as the proportion of valid medication-reporting days with any rescue medication in each principal window. The paired contrast is post-treatment minus pretreatment and will be reported in percentage points.

For dysmenorrhoea, participant-level means will be calculated across assessable diary days in each principal window among participants with at least one valid dysmenorrhoea rating in both windows. Days on which dysmenorrhoea is not assessable are not coded as zero.

5.4 Patient-reported and psychophysical measures

RAND 36-Item Health Survey 1.0 domain scores will be derived on a 0–100 scale using the standard item recoding and domain definitions, with higher scores indicating better health. Patient Global Impression of Change (PGIC) responses of “much improved” or “very much improved” will define a favourable response. The endometriosis-associated symptom selected as most troublesome at baseline will remain fixed at follow-up; if the relevant event has not occurred, its impact score will be recorded as not assessable rather than zero.

For pressure-pain threshold (PPT), three ascending measurements at each site will be averaged. The bilateral rectus-abdominis site means will then be averaged to define local abdominal PPT; the right tibialis-anterior site mean will define remote PPT. Units are kilopascals.

5.5 Gynaecological phenotype

A single primary endometriosis-confirmation source will be assigned for descriptive reporting in the following order: histology, operative documentation, expert transvaginal ultrasonography and magnetic resonance imaging. Definite adenomyosis will require at least one direct revised-MUSA feature; examinations showing indirect features only will be classified as indeterminate, and those showing

neither direct nor indirect features as absent. Compartments recorded as not assessable will be excluded from the relevant #Enzian denominator rather than coded as disease-free.

6. General statistical principles

Continuous variables will be summarised as mean and standard deviation (SD) when a mean-based summary is appropriate and as median and interquartile range (IQR) for skewed durations and other prespecified implementation measures. Categorical variables will be reported as counts and percentages with explicit denominators. Ranges will be added where clinically or operationally informative. Baseline characteristics will not be compared using null-hypothesis significance tests.

All confidence intervals will be two-sided and reported at the 95% level. A nominal two-sided alpha level of 0.05 will be used for the primary and key secondary model contrasts. Interpretation will emphasise effect magnitude and precision. Daily pelvic pain is the sole primary outcome and daily activity interference is the key secondary outcome. All other analyses are supportive or exploratory; no formal multiplicity adjustment will be applied to those analyses.

Changes are defined as follow-up minus pretreatment. Negative values therefore indicate improvement for pain, activity interference, dysmenorrhoea and symptom-impact ratings, while positive values indicate improvement for RAND-36 and PPT measures.

7. Participant flow and baseline description

Participant flow will be reported from pathway entry through clinical indication, research eligibility, invitation, consent, treatment initiation and inclusion in the outcome-specific analysis sets. Reasons for non-indication, research ineligibility, non-participation, non-initiation and follow-up noncompletion will be reported using the denominator relevant to each transition.

Baseline characteristics will be summarised for the enrolled cohort and according to inclusion versus exclusion from the primary pain longitudinal set. PHQ-9 and GAD-7 total scores and expected pain reduction will be treated as baseline descriptive measures. Standardised mean differences (SMDs), rather than significance tests, will be used to describe differences between these groups. For a continuous variable, the SMD will use the difference in means divided by the square root of the average of the two group variances. Binary characteristics will use the difference in proportions divided by the corresponding pooled binomial standard deviation. SMDs will be interpreted descriptively.

8. Primary and key secondary longitudinal analyses

8.1 Primary daily pelvic pain model

The primary model includes daily pain observations from the pretreatment and post-treatment principal windows. Pretreatment and post-treatment are modelled as separate phases. Within each phase, day is represented by a natural cubic spline with three degrees of freedom. Participant-specific random effects comprise a random intercept and a random post-treatment phase effect, fitted with an unstructured 2×2 covariance matrix. Residual serial dependence is represented by a continuous-time first-order autoregressive correlation structure based on the absolute difference between actual diary dates.

Variance parameters will be estimated by restricted maximum likelihood (REML). The principal contrast is the difference between the marginal mean averaged across scheduled post-treatment days 1–28 and the corresponding marginal mean averaged across the 28 pretreatment days. The contrast is

therefore a phase-average difference rather than a single-day endpoint. The principal contrast will be reported with a 95% confidence interval and a two-sided P value.

Treatment-phase observations will not enter this model and will be shown descriptively as observed daily means.

8.2 Key secondary daily activity-interference model

Daily activity interference will be analysed in its outcome-specific longitudinal set using the same phase definition, spline specification, random-effects structure, continuous-time residual correlation, REML estimation, phase-average contrast and inferential procedure as the primary pain model. The analysis is separate from the pain model and uses all eligible observed interference ratings.

8.3 Prespecified sensitivity analyses

Four sensitivity analyses will be performed separately for pain and activity interference. Except for the change stated below, the model specification and principal 28-day phase-average contrast remain unchanged.

**Sensitivity analysis**	**Prespecified modification**
Daily menstrual bleeding	Add daily menstrual-bleeding status as a fixed covariate. Rows with missing bleeding status do not contribute to this sensitivity model.
Diary adherence	Restrict to participants with at least 80% outcome-specific diary completion in both principal windows.
Per protocol	Restrict to the prespecified per-protocol set.
Major new pain-directed intervention	Exclude participants who initiate a major new pain-directed intervention before post-treatment day 28.

Sensitivity analyses will be interpreted from the point estimates and 95% confidence intervals without separate inferential emphasis.

8.4 Model-independent participant-level estimate

For pain and activity interference, a participant-level mean will also be calculated in each principal phase. The mean paired change across participants will be reported with a t-based 95% confidence interval. These participant-level pretreatment and post-treatment means will provide a model-independent secondary estimate.

9. Supportive and exploratory analyses

9.1 Week-8 diary outcomes

Week-8 pain and activity interference will be analysed separately in the corresponding week-8 outcome set. A participant-level mean will be calculated for pretreatment and for the valid week-8

diary days. Change will be estimated as the paired mean difference, with a t-based 95% confidence interval. Week-8 observations will not be added to the primary or key secondary longitudinal models.

9.2 Pain responders

The proportions achieving at least 30% and at least 50% pain reduction will be reported in the pain responder set. Wilson 95% confidence intervals will generally be used, with exact binomial intervals reserved for sparse counts. Participants with insufficient diary data will not be assigned responder status but may still contribute to the longitudinal pain model if its eligibility criteria are met.

9.3 RAND-36 and PGIC

Each RAND-36 domain will be analysed using a linear mixed-effects model with categorical visit (baseline, week 4 and week 8) as a fixed effect and a participant-specific random intercept. Changes from baseline at weeks 4 and 8 will be reported with 95% confidence intervals. These domain analyses are supportive and no multiplicity adjustment will be applied.

PGIC will be summarised at weeks 4 and 8 as the proportion reporting “much improved” or “very much improved”. Wilson 95% confidence intervals will generally be used, with exact binomial intervals reserved for sparse counts.

9.4 Participant-selected symptom, dysmenorrhoea and rescue medication

Impact-score changes at weeks 4 and 8 will be estimated as paired mean differences among participants with assessable responses at baseline and at the relevant follow-up. The same symptom selected at baseline must be used at each follow-up. T-based 95% confidence intervals will be reported.

Dysmenorrhoea will be analysed as the paired difference between participant-level pretreatment and post-treatment means among participants with at least one assessable rating in both windows. Rescue-medication use will be analysed as the paired difference in the proportion of valid medication-reporting days with any use. Both analyses will use t-based 95% confidence intervals and will be interpreted as supportive.

9.5 Pressure-pain thresholds

Local abdominal PPT and remote tibialis-anterior PPT will be analysed separately as baseline-to-week-4 paired mean differences among participants with valid measurements at both assessments. Paired mean differences will be reported with t-based 95% confidence intervals; bootstrap confidence intervals will be used if the distribution of within-participant differences is markedly non-normal.

9.6 Exploratory adenomyosis modifier

The only prespecified clinical modifier analysis will compare participants with definite versus absent adenomyosis; indeterminate examinations will be excluded. The primary pain model will include adenomyosis group and an adenomyosis-by-phase interaction while retaining the prespecified within-phase time structure. The principal exploratory quantity is the difference in the 28-day phase-average pain change between definite and absent adenomyosis. The interaction estimate and 95% confidence interval will be reported without definitive subgroup inference.

10. Implementation, treatment fidelity, safety and acceptability

Implementation summaries will include progression through the clinical pathway, intervals from clinical indication to research screening, research screening to session 1, and clinical indication to session 1, treatment initiation, treatment-course span, follow-up completion and diary adherence.

Treatment-course span is the elapsed number of calendar days between the first and final delivered sessions and will be summarised among participants who attend all ten scheduled sessions.

Treatment exposure and fidelity will be summarised in the treated cohort and at the session level. Participant-level measures include number of sessions attended, attendance at all ten sessions and at least eight sessions, cumulative pulse dose, missed-session reasons and the presence of authorised modifications or major deviations. Session-level categories are: complete protocol-concordant; complete with an authorised modification; incomplete without a major dose deviation (1,200–1,499 pulses); and major deviation (<1,200 pulses or an unauthorised major treatment-parameter change). Procedure and total visit durations will be summarised as mean and SD.

Safety analyses will use the treated cohort. A treatment-emergent adverse event (TEAE) is any unfavourable condition first appearing or worsening after the first therapeutic pulse through week 8, irrespective of causality. TEAEs will be summarised by participant and episode, with event type, severity, seriousness and resolution reported descriptively. Treatment discontinuations due to TEAEs will also be reported. The proportion of participants with at least one TEAE and the episode rate per 100 delivered sessions will be reported with 95% confidence intervals. A persistent unchanged event will be counted once unless it worsens or requires new action.

Acceptability will be described among treated participants with an available week-4 assessment. Satisfaction on the 0–10 scale will be summarised as mean ± SD. The responses to whether the participant would choose the same treatment again will be reported as yes, unsure or no, with explicit denominators; reasons for negative or uncertain responses will be summarised descriptively.

11. Missing data and intercurrent events

Daily pain and activity-interference ratings will not be singly imputed. The longitudinal mixed-effects models will use all eligible observed outcome values under a missing-at-random assumption conditional on the fitted model. Minimum diary requirements define the analysis sets; participants who do not meet those requirements will be excluded only from the analysis for which the requirement applies.

Missing paired follow-up observations for week-8 diary outcomes, symptom impact, dysmenorrhoea, rescue medication and PPT will not be imputed. Diary completeness and reasons for follow-up noncompletion will be reported. Baseline characteristics of participants included in and excluded from the primary longitudinal set will be compared descriptively using SMDs.

Treatment discontinuation, authorised modifications, protocol deviations and medically necessary changes in concomitant care will not censor subsequent outcome observations. Participants will remain eligible for follow-up after treatment discontinuation. A major new pain-directed intervention before post-treatment day 28 will be addressed by the prespecified exclusion sensitivity analysis rather than by censoring the principal analysis.

12. Model diagnostics

For the primary and key secondary models, convergence and residual diagnostics will be reviewed. If a prespecified longitudinal model cannot be fitted as intended, the reason and any alternative specification used will be documented.

13. Data quality and software

Before final analysis, range, date and internal-consistency checks will be completed and outstanding data queries resolved. Analyses will be performed in R (R Foundation for Statistical Computing). The software version used for the final analysis will be recorded.

14. Deviations from this plan

Any departure from this plan will be documented with its rationale and timing relative to review of outcome results. Additional analyses not specified here will be identified as exploratory or post hoc.
